# “I Might Need This for Later.” “Back Pocketing” Hospice Information among Caregivers of Black PLwD

**DOI:** 10.1093/geroni/igaf122.3694

**Published:** 2025-12-31

**Authors:** Emika Miller, Karen Moss, Karen Rose, Seuli Brill, Mary Beth Happ

**Affiliations:** Ohio State University College of Nursing, Fairborn, Ohio, United States; The Ohio State University, Blacklick, Ohio, United States; The Ohio State University, The Ohio State University, Ohio, United States; College of Medicine, Ohio State University, Ohio, United States; Ohio State University, Columbus, Ohio, United States

## Abstract

Despite the benefits associated with hospice (e.g., improved quality of life at end-of-life), Black people living with dementia (PLwD) are less likely to utilize hospice services compared to non-Hispanic White persons. Exposure to hospice information may impact end-of-life decision-making for Black PLwD. This qualitative descriptive study examined the sources and characteristics of hospice information among caregivers and its influence on hospice decision-making. We employed semi-structured interviews with family caregivers (N = 25) of deceased Black PLwD. Longo’s Expanded Model guided the initial coding process. Thematic narrative analysis was applied to coded data through an iterative process. The study sample was predominantly female (72%), with an average age of 55.5 ± 14.7 years, and served as caregivers for 5.0 ± 4.0 years. Most participants (75%) enrolled their PLwD in hospice. When encountering dementia, hospice or other health-related information, some participants chose to “back pocket” it. “Back pocketing” refers to saving information for later consideration and possible action. Reasons for “back pocketing” information included: 1) deeming the information or source questionable in credibility, or 2) providing a knowledge bank to “pull from” for future decision-making in their caregiving role. One participant found “back pocket” information instrumental in securing a dementia diagnosis for their PLwD. This study refutes the notion that Black people do not receive or retain hospice information. It also emphasizes the importance of providing caregivers with credible hospice information and asking caregivers about “back pocketed” information. These findings may assist clinicians in determining when and through which avenues to provide hospice-related information.

